# The Influence of Zusanli and Nonmeridian Acupuncture Points on the Survival Rate and Intestinal Tissue Features after Fatal Hemorrhagic Shock in Rats

**DOI:** 10.1155/2013/750620

**Published:** 2013-01-30

**Authors:** Xian Shi, Yuxian Zhong, Jiarui Yao, Sen Hu, Lu Wang, Gerhard Litscher

**Affiliations:** ^1^Department of Acupuncture, People's Liberation Army General Hospital, 28 Fu-Xing Road, Beijing 100853, China; ^2^Department of Rehabilitation and Physiotherapy, Navy General Hospital, Beijing 100048, China; ^3^Laboratory of Shock and Multiple Organ Dysfunction, Burns Institute, The First Hospital Affiliated to the People's Liberation Army General Hospital, Beijing 100037, China; ^4^Stronach Research Unit for Complementary and Integrative Laser Medicine, Research Unit of Biomedical Engineering in Anesthesia and Intensive Care Medicine and TCM Research Center Graz, Medical University of Graz, Auenbruggerplatz 29, 8036 Graz, Austria

## Abstract

Sixty Sprague-Dawley rats were divided into 5 groups: (a) control group (HS); (b) Immediate rehydration group (IFR); (c) ST36 electroacupuncture (EA) delay rehydration group (EA/DFR): EA at ST36 immediately after blood loss with infusion 3 h later; (d) EA nonmeridian rehydration group (SEA/DFR): EA at nonacupuncture sites with rehydration similar to EA/DFR; (e) ST36 EA group (EA): EA at ST36 immediately after blood loss with no rehydration. Forty-five percent of the entire blood volume was taken out to make lethal hemorrhagic shock models. We recorded the survival rate, intestinal tissue DAO content, and microcirculation. The survival rate of the EA/DFR group and the IFR group was significantly higher than that of the other three groups (*P* < 0.05). Twelve hours after blood loss, intestinal tissue DAO content of the EA/DFR group and the IFR group was significantly higher than that of the SEA/DFR group, EA group, and HS group (*P* < 0.05 and *P* < 0.01). The mucosal blood flow of the EA/DFR group and the IFR group was significantly higher than the other groups (*P* < 0.05 each). We conclude that EA improves the blood pressure and raises the early survival rate of hemorrhagic shock rats, maintains the intestinal barrier function, and improves the degree of intestinal ischemia.

## 1. Introduction

Nowadays, accidents, wars, and disasters occur frequently. In a short period of time, there can be a batch of wounded in hypovolemic shock. Conventional antishock means (such as blood transfusion, or infusion) are difficult to implement timely. The prolonged hypoperfusion will cause damage to the intestinal mucosal barrier of the body, increase the systemic inflammatory response, and induce sepsis, which increases mortality or complications [1]. The acupoint ST36 (Zusanli) on the stomach meridian is considered to be the main point of regulation of gastrointestinal function, promoting gastrointestinal peristalsis and detoxification and protecting the mucosal barrier [2]. Therefore, we studied the mechanism of the ST36 acupuncture point in fatal hemorrhagic shock in rats.

## 2. Materials and Methods

### 2.1. Sprague-Dawley Rats

Sixty SPF (specific pathogen free) Sprague-Dawley male adult rats (weight 270 ± 25 g) were kept under constant temperature (24 ± 2°C) and constant humidity of 50 ± 5% for one week. They had to refrain from eating for 12 hours and were forbidden to drink for 4 hours preoperatively. The study was approved by the Institutional Animal Care and Use Committee (license number SCXK Beijing 2009/0007) and was in accordance with National Institutes of Health guidelines. The animals were anesthetized using an intraperitoneal injection of ketamine hydrochloride and sumianxin new II (0.4 mL/kg, mixed 2 : 1 by volume).

### 2.2. Monitoring and Stimulation Equipment

For monitoring the microcirculation of intestinal tissue, we used a Laser Doppler flowmetry system (PeriFlux5000, PERIMED, Jarfälla, Sweden). The detection of diamine oxidase (DAO) was performed using kits from Nanjing Jiancheng Technology Co.; the micropump for infusions is a product of Millipore Corporation, Japan. The electroacupuncture (EA) stimulator was an INTI KWD-808I-type pulse meter from Changzhou Yingdi Electronic Medical Device, Changzhou, China.

### 2.3. Experimental Procedure

Based on the improved method described in the publication of Higashimura et al. [3], we used an animal model with 45% blood loss. The rats underwent femoral vein dissection and catheterization; femoral artery dissection and catheterization; and carotid dissection and intubation at the neck incision. Then a cardiopulmonary capacity monitor was connected to monitor mean arterial pressure (MAP). The abdominal cavity was cut open along the ventral midline about 5 cm, and laser Doppler probes were placed 10 cm from the Treitz ligament. A 1% heparin saline solution was injected into the femoral vein to make the animals systemically heparinized. After this was completed, the time was metered −0.5 h, that is, 30 min before blood loss. Then, bloodletting from the femoral artery started. First, 30% of the total blood volume was taken out at a constant rate in 15 min. Then bleeding was stopped for 5 min, before another 15% of the total blood volume was taken out at a constant rate in 10 min. So, the entire process was completed in 30 min, and the total blood volume taken out was 45%. The rat systemic estimated blood was calculated using the following formula: estimated systemic blood = weight (g) × 0.06 (mL/g) + 0.77 [3]. The completion of the hemorrhagic shock model concluded the preparation phase, and the time was metered 0.

All animals were observed at four time points before blood loss and after hemorrhagic shock: 30 min before blood loss (−0.5 h); 3 h, 12 h, and 24 h after blood loss. The rats were randomly divided into five groups (12 animals each).

- Hemorrhagic shock group (HS) for control purposes; this group did not receive any intervention (acupuncture or rehydration treatment).
- The immediate rehydration (IFR) group was given a femoral vein infusion with Ringer lactate (2 times the amount of blood loss) immediately after blood loss, lasting 40 min.
- The electroacupuncture (EA) group received EA bilaterally at the ST36 point on the stomach meridian. EA was done immediately after blood loss; needle insertion depth 7 mm [4], stimulation for 40 minutes, frequency 4 Hz, constant voltage 4 V. The animals in this group did not receive rehydration.
- In the EA/DFR (delayed rehydration) group, animals were infused 3 h after blood loss; the infusion method and liquid were the same as the IFR group, and EA parameters were the same as in the EA group.
- The SEA/DFR group was electro-acupunctured at nonacupoints (0.5 cm lateral and distal from ST36, i.e., not on the meridian) immediately after blood loss; other interventions were the same as in the EA/DFR group.

### 2.4. Sample Collection and Processing

- Index determination
- The surviving rats in each group were counted 3 h, 12 h, and 24 h after blood loss for statistical analysis;
- MAP was recorded using a cardiorespiratory monitor;
- monitoring microcirculatory blood flow of intestinal mucosal tissue was performed with a laser Doppler probe and measured three times to obtain an average value
- 100 mg of the small intestine tissue were retrieved at a distance of 5 cm from the cecum. We added 0.9 mL of 0.9% saline solution to grind, homogenizate, and obtain the supernatant. A DAO kit and spectrophotometer was used to measure the activity of DAO.

### 2.5. Statistical Analysis

SPSS 17.0 statistical software was used to calculate percentages, means, and standard deviations. One-way ANOVA was used for comparison among all groups, followed by the Student-Newman-Keuls (SNK) test for comparison between two groups. *P* < 0.05 was defined as the level of statistical significance.

## 3. Results

### 3.1. Survival Rate

**Figure 1:**
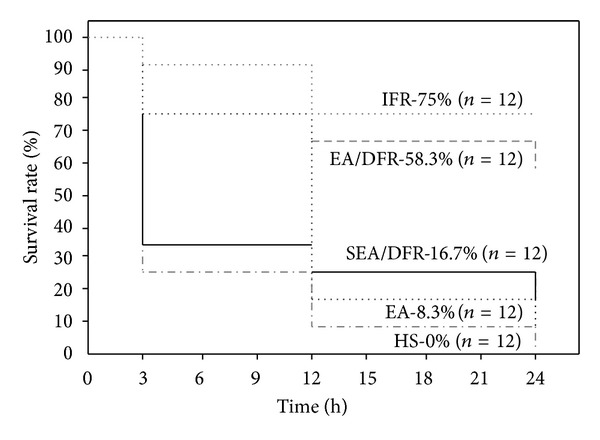
Kaplan-Meier graph of survival rate of rats in each group after 45% blood loss. *N* = 12 refers to the original number of animals in each group.

The survival rate of the EA/DFR group, EA group, and IFR group was significantly higher than that of the HS group at the point of 3 h (*P* < 0.05 each). At the point of 12 h and 24 h, the survival rate of the IFR group and the EA/DFR group was significantly higher than that of the other groups (*P* < 0.05 each) (see Figure 1).

### 3.2. Mean Arterial Blood Pressure (MAP)

**Figure 2:**
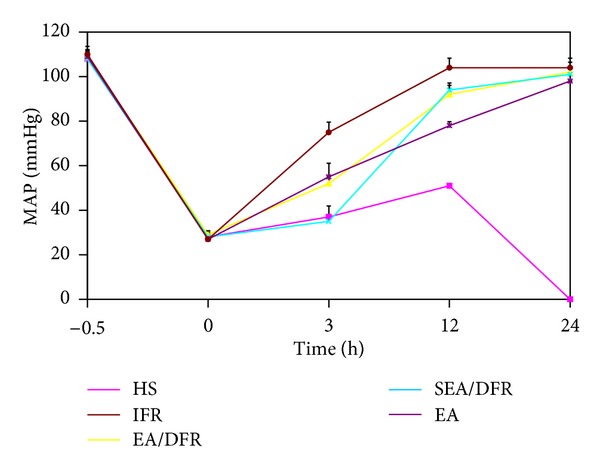
The change of MAP (mean ± SE) at different time points after blood loss.

MAP in each group was significantly lowered after blood loss. MAP at time 0 was only 25%-26% of that before blood loss; after 3 hours, MAP in each group increased by different degrees. MAP of the IFR group was significantly higher than that of the other groups (*P* < 0.01 each), and MAP of the EA group and the EA/DFR group was significantly higher than that of the SEA/DFR group (*P* < 0.01 each). After 24 h, there was no significant difference in MAP between the groups (Figure 2).

### 3.3. DAO Activity Changes in Intestinal Tissue

Three hours after blood loss, the DAO content of intestinal tissue in each group was significantly lower compared to the point before blood loss. The activity of the IFR group was significantly higher than that of the other four groups (*P* < 0.05 each); however, the EA/DFR group and EA group were significantly higher than the SEA/DFR group and the HS group (*P* < 0.05 each). Twelve hours after blood loss, the IFR group was significantly higher than the other four groups (*P* < 0.05 each), but the EA/DFR group was significantly higher than the HS group, SEA/DFR group and EA group (*P* < 0.01 each) (see Table 1).

### 3.4. Mucosal Blood Flow

After blood loss, mucosal blood flow in each group was significantly lowered (*P* < 0.05 each). Three hours after blood loss, the mucosal blood flow of the EA/DFR group and the EA group was lower than that of the IFR group (*P* < 0.05), but significantly higher than the HS group and the SEA/DFR group (*P* < 0.05 each). Twelve hours after blood loss, there was no significant difference between the EA/DFR group and the IFR group (*P* > 0.05), but these two groups were significantly higher than all other groups (*P* < 0.05 each) (see Table 2).

## 4. Discussion

In order to ensure the blood supply of the vital organs during hemorrhagic shock, a sharp reduction in the intestinal blood flow and in the intestinal mucosal functional barrier occurs under the action of the sympathetic-adrenal medullary system and vasoconstrictor substances, such as catecholamine. Hence intestinal endotoxemia and bacterial translocation act on the liver via the portal vein through the portal circulation and lymphatic system. Endotoxin can cause a lack of the nutrition bloodstream to the liver and lower the degree of mitochondrial oxygen metabolism, which may cause liver dysfunction or even liver failure. Liver dysfunction can accelerate the diffusion of endotoxin in vivo, which can lead to multiple organ dysfunction syndrome (MODS) or even death. All the processes mentioned above are important reasons which make hemorrhagic shock irreversible [1], and they are also important factors for the high mortality rate of hemorrhagic shock. Therefore, maintaining bowel function and protecting the mucosal barrier can delay the development of hemorrhagic shock and avoid the transition to septic shock; furthermore it can improve delay rehydration effect and the survival rate of patients with hemorrhagic shock [5, 6]. 

Our previous research [7, 8] indicates that the ST36 acupuncture point is capable of activating the cholinergic anti-inflammatory pathway, as the result of reducing the level of rats' intestinal tissue proinflammatory cytokine and intestinal tissue edema and dysfunction.

The present study shows that the ST36 acupuncture point improves rehydration, rats' blood pressure levels, and the subsequent rehydration treatment effect to a certain extent, which increases the survival rate of rats in hemorrhagic shock. At the same time, it shows a significant change of the DAO content of intestinal tissues after massive blood loss. DAO is an enzyme which is present in the small intestine mucosa upper villus cell in mammalians. It can reflect the small intestine mucosal structure and function [9]. When hemorrhagic shock occurs, the function of the intestinal barrier is damaged, and DAO of the intestinal mucosa is released into the blood, which leads to an increase of DAO activity in plasma and a decrease of DAO activity in intestinal tissue [10]. However, when acupuncturing the ST36 point, the content of DAO and the blood flow of the intestinal mucosa were significantly higher than those of the non-electroacupuncture group and the sham acupuncture group. This illustrates that the ST36 acupuncture point has a protective effect on intestinal mucosa. 

In conclusion, only acupuncture at the ST36 point, located on a meridian, can improve the survival rate of rats with fatal hemorrhagic shock, which may have a direct relationship with the improvement of the intestinal barrier's function and an increase of blood flow.

## Figures and Tables

**Table 1:** The change of DAO activity of intestinal tissue (mean ± SD) at different time points after blood loss.

Group	*N* (originally)	DAO [U/L]		
		−0.5 h	3 h	12 h
HS	12	48.5 ± 2.7	20.1 ± 2.2^ab^	—
IFR	12	49.3 ± 3.9	38.1 ± 1.6^b^	44.1 ± 2.8^b^
EA/DFR	12	48.8 ± 3.0	29.9 ± 4.5^a^	37.9 ± 3.4^a^
SEA/DFR	12	49.2 ± 1.1	20.5 ± 2.6^ab^	30.3 ± 1.3^ab^
EA	12	48.3 ± 1.6	28.6 ± 2.8^ab^	—

Note: the comparison with IFR group, ^a^ *P* < 0.05; the comparison with EA/DFR group, ^b^ *P* > 0.05. “—” means that the number of surviving animals was too small to calculate statistical values.

**Table 2:** The monitoring results of changes in mucosal blood flow (mean ± SD) in each group.

Group	*N* (originally)	The intestinal mucosal blood flow [perfusion unit (PU)]		
		−0.5 h	3 h	12 h
HS	12	211.3 ± 21.9	85.6 ± 11.8^ac^	—
IFR	12	209.9 ± 27.1	152.8 ± 23.6	149.6 ± 10.8
EA/DFR	12	216.8 ± 50.9	116.1 ± 16.6^a^	151.4 ± 21.4^b^
SEA/DFR	12	212.4 ± 49.5	88.7 ± 13.1^ac^	102.3 ± 27.2^ac^
EA	12	212.8 ± 36.7	115.8 ± 14.2^a^	—

Note: the comparison with IFR group, ^a^ *P* < 0.05, ^b^ *P* > 0.05; the comparison with EA/DFR group, ^c^ *P* < 0.05. “—” means that the number of surviving animals was too small to calculate statistical values.
